# D-Dimer: A Potential Solution to Problems of Cancer Screening, Surveillance, and Prognosis Assessment

**DOI:** 10.7759/cureus.15064

**Published:** 2021-05-16

**Authors:** Nabeel A Siddiqui, Mushrin Malik, Ransirini Wijeratne Fernando, Archana Sreekantan Nair, Janan Illango, Rajvi Gor, Pousette Hamid

**Affiliations:** 1 Research, California Institute of Behavioral Neurosciences & Psychology, Fairfield, USA; 2 Neurology, California Institute of Behavioral Neurosciences & Psychology, Fairfield, USA

**Keywords:** d-dimer, cancer, malignancy

## Abstract

Research has established a direct link between the plasma level of D-dimer and underlying malignancy. D-dimer has a strong association with the detection and prognosis of several cancers. For these reasons, this literature review aimed to evaluate the usefulness of elevated D-dimer levels in the initial screening of cancer, cancer recurrence surveillance, and for use as a cancer prognostic tool. A search of PubMed up to February 1, 2021, was carried out by reviewers. This literature review includes studies investigating the relationship between pretreatment plasma D-dimer levels and cancer. From the findings, pretreatment D-dimer levels can assist with cancer screening and prognosis assessment. Pretreatment plasma D-dimer levels can function as an effective cancer recurrence control. Elevated pre-treatment plasma D-dimer concentration is valuable in facilitating cancer screening, predicting an augmented risk of cancer recurrence, and anticipating a worse cancer prognosis.

## Introduction and background

About 39.5% of people will have cancer at some point in their life [1]. Estimated national spending on cancer treatment in the United States in 2018 was $150.8 billion [1]. Costs will escalate in coming years as the population ages and more people get cancer. Costs will further escalate as modern and costly procedures become embraced as quality care. Cancer has an enormous negative impact around the world. Early detection of cancer and cancer recurrence via advanced cancer screening methods can help treat cancer more effectively and reduce national spending. Human cells typically expand and divide when the body requires them to creating new cells. Cells typically die as they get old, and new cells take their place. This orderly process breaks down as cancer arises. Cancer creates dysfunctional cells, which continue to grow and divide when they should die. Without stopping, these extra cells can form growths called tumors.

**Figure 1:**
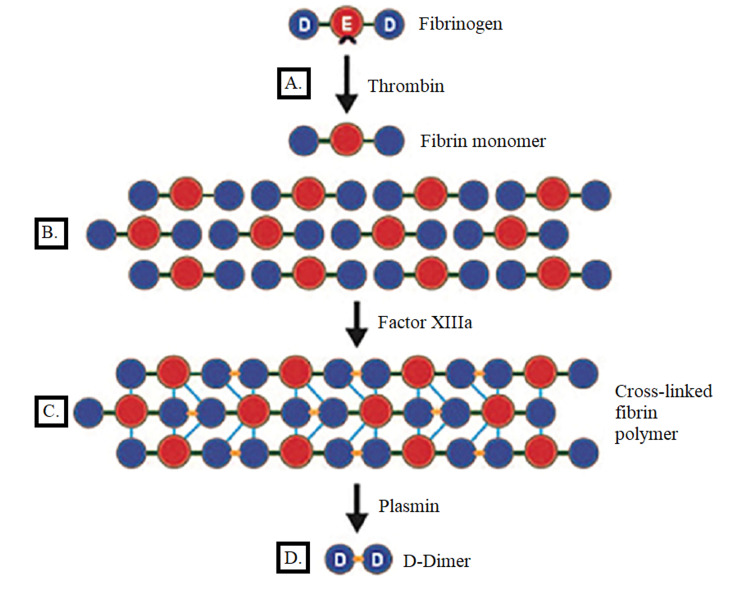
Generation of D‐dimer following thrombin generation and fibrinolysis A. Thrombin forms fibrin monomers by cutting fibrinopeptides from the fibrinogen E-domain. B. Fibrin monomers aggregate. C. Fibrin monomers become cross‐linked by factor XIIIa to produce a fibrin clot. D. The breakdown of cross‐linked polymers by plasmin results in the release of fibrin degradation products, including D‐dimer.

D-dimer molecules are produced by the breakdown of cross-linked fibrin during fibrinolysis. D-dimer generation requires the activity of thrombin, active factor XIII (factor XIIIa), and plasmin. The process begins when the thrombin formed by the coagulation system transforms soluble fibrinogens into fibrin monomers. As a result of thrombin fibrinopeptide cleavage from the N-terminal domain, these monomers form fibrin polymers dependent on allosteric protein modifications. Fibrin becomes reinforced through interactions with factor XIII, which, after activation by thrombin, cross‐links the D domains of neighboring fibrin monomers. Plasmin degradation of the fibrin clot results in the D‐dimer molecule (Figure 1).

D-dimer is a biomarker that is routinely tested to screen for deep venous thrombosis (DVT), disseminated intravascular coagulation (DIC), and pulmonary embolism (PE) in clinical practice. D-dimer is a universally available, regularly utilized, and essential molecular marker. D-dimer is rapidly removed from the human body, which leads to a low basal level of d-dimer, resulting in an enhanced sensitivity. Elevated levels of D-dimer indicate the activation of coagulation and fibrinolysis [2]. In patients with coronary artery disease, trauma, pregnancy, and inflammatory disorders, circulating D-dimer may also be elevated. One of the most common complications associated with cancer is developing a coagulation disorder [2], and irregularities of coagulation and fibrinolysis are often observed in cancer patients [3]. Elevated D-dimer levels have been observed in the plasma of patients with several solid cancers, including the prostate [4], cervix [5], and esophageal squamous cells [6]. Plasma D-dimer was also noted to be markedly elevated in patients with various malignancies, including lung cancer [7], prostate cancer [8], cervical cancer [5], breast cancer [9], and colorectal cancer [10].

The association between D-dimer levels and cancer progression remains to be a focus of study. To this day, the association between D-dimer and cancer is unclear. The latest findings have demonstrated a possible association between coagulation, fibrinolysis, and tumor prognosis. The value of serum D-dimer levels as a prognostic marker remains undefined. Some have stated that an elevated pretreatment D-dimer level is associated with a more advanced tumor stage or a more advanced clinical progression, which suggests a worse prognosis. However, others believed that this relationship was negligible. For now, only a few attempts have been made to examine the accuracy and magnitude of D-dimer level prognostic importance systematically. Establishing cancer diagnosis as early as possible can help provide early treatment to patients, which is essential to improving survival rates. Clinical laboratory indicators are immediately desirable to assist in the differential diagnosis and predict prognosis.

Coagulation caused by tumors is closely related to the growth of tumors. Years before the patient has any apparent clinical symptoms; malignant diseases may display signs of venous thromboembolism [11]. A vicious cycle between procoagulant proteins and malignant tumor cells is created by promoting neovascularization and metastasis [12]. There is evidence that activated fibrinogens prevent the removal of natural killer cell-mediated tumor cells, enhance the survival of circulating tumor cells, increase the risk for tumor metastases, and contribute to poor prognosis [13]. Therefore, D-dimer levels have clinical value for predicting prognosis and differential screening of benign and malignant tumors [14].

Studies have shown that D-dimer has a significant correlation with the diagnosis and prognosis of various malignant tumors. Current literature shows that D-dimer levels may be an effective tool to use as a diagnostic marker and to design more individualized and effective treatment strategies. The current cancer prognostic evaluation process has its disadvantages, whereby patients with the same TNM level often have different survival outcomes. D-dimer can serve as an external predictor that could help to improve the estimate of the prognosis. Furthermore, dysregulation of the clotting system may be the first indication of tumor cell proliferation. For this reason, D-dimer may be useful in the screening of cancer or to monitor cancer recurrence. Ultimately, the measurement of plasma D-dimer levels is a non-invasive and affordable test that may be useful in the initial screening of cancer, cancer recurrence surveillance, and as a cancer prognostic tool.

High-quality studies evaluating the association between D-dimer levels and cancer are currently limited. Numerous studies have assessed the role of clotting activation biomarkers in cancer outcomes, including disease progression, response to treatment, and mortality. However, many of these studies were retrospective and were not precisely designed to address the impact of D-dimer on cancer outcomes. Moreover, recruitment was often mono-institutional and included small heterogeneous cohorts of patients with various therapies. For this reason, we included several studies with low-quality evidence and did not conduct any critical assessments of the studies reviewed. In the present literature review, we provide a superficial overview that is meant to highlight possible oncological care advancements and encourage the research community to conduct more in-depth research on this topic. Despite the promising results reported, new data from large prospective cohorts are needed.

## Review

Role of D-dimer in cancer screening and as a marker for cancer recurrence

In patients with breast, prostate, gynecologic, and lung cancers without clinical thrombosis, elevated D-dimer levels have been reported [15]. D-dimer levels were most elevated in patients with pancreatic cancer and lowest in prostate cancer patients. Even so, Korean authors observed that D-dimer levels were substantially higher in patients with prostate cancer compared to patients without prostate cancer at prostate biopsy [16]. In another study, investigators found that patients with advanced prostate cancer had a substantial rise in D-dimer levels relative to age-matched controls and patients with localized prostate cancer. Further studies are needed to establish a relationship between the coagulation process and prostate cancer and to validate the utility of the plasma D-dimer level as a marker for prostate cancer. 

Connections between D-dimer and the clinical biology of breast cancer have been suggested in recent years. The findings indicated a significantly higher D-dimer level in the breast cancer community than in the benign and healthy control groups. Increased plasma D-dimer levels indicate increased activation of the coagulation system in breast cancer patients, indicating that the level of plasma D-dimer may have a supplementary benefit for clinical breast cancer diagnosis. Studies have shown that D-dimer sensitivity and specificity are more significant than that of the current cancer antigen 15-3 and carcinoembryonic antigen (CEA) tumor markers [17]. Unfortunately, much of the study data did not allow for calculations of sensitivity and specificity of the D-dimer level's effect indicator for breast cancer diagnosis.

Blood clotting activation can be an overt indicator of occult cancer, confirmed by clinical trials of cancer-free subjects, which indicate that hypercoagulability is a risk factor for eventual death from cancer [18]. Recurrence may result from cancer cells that remain in the host following chemotherapy and continue to proliferate and spread after years of inactivity. Dysregulation of the clotting system may be the first indication of tumor cell proliferation, and elevated thrombotic biomarkers like D-dimer may help assess this dysregulation.

Chen et al. reported that according to their Retrospective Cohort Study data, elevated plasma D-dimer levels predicted an increased risk of cancer recurrence and a worse prognosis in patients diagnosed with Upper Tract Urothelial Carcinoma, which is a rare urinary tumor with an unfavorable prognosis [19]. Their data found that plasma D-dimer levels ≥ 0.36 mg/L correlated with higher pathological T stage, tumor grade, and invasion of cancer to the blood vessels or lymphatics. Their results suggest that preoperative plasma D-dimer levels are an essential biomarker for predicting oncological outcomes, including cancer recurrence in Upper Tract Urothelial Carcinoma patients.

Role of D-dimer as a prognostic tool for cancer

Cancer prognosis is always associated with metastases, a mechanism involving multiple tumor-host interactions. Metastatic cancer cells must exit the primary tumor site and spread through the lymphatic or vascular system. These cancer cells eventually create a new blood supply. Fibrin remodeling is almost always involved in the process of metastases and has been shown to provide a vital role in the formation of new blood vessels [20]. The tissue factor is a crucial agent in activating the extrinsic pathway and plays a vital role in cancer metastases and progression. Consequently, elevated D-dimer levels could be the result of abnormally active tissue factor.

Distant metastasis is the primary cause of poor prognosis and contributes to inefficiency in cancer patients' treatment [21]. The latest findings have demonstrated a clear correlation between circulating tumor cells and plasma D-dimer levels in patients with metastatic breast cancer [22]. Many tumor-associated coagulation factors, including the tissue factors, fibrin, and plasmin, are dysregulated during tumor growth, metastasis, thrombosis, and angiogenesis in malignancy [23]. The elevated levels of plasma D-dimer reflect the dysregulation between coagulation and fibrinolysis; therefore, preoperative plasma D-dimer levels might help assess the prognosis of patients diagnosed with cancer.

Ay et al. conducted a prospective and observational cohort study evaluating the prognostic value of D-dimer levels with the overall survival (OS) and mortality risk in 1,178 cancer patients [24]. They indicated that elevated D-dimer levels were associated with an increased risk of death in patients with lymphomas, brain tumors, pancreatic, prostate, breast, lung, stomach, and colorectal cancers. This study shows the importance of D-dimer levels and their correlation with low OS and increased risk of death in cancer patients.

Li et al. conducted a systematic review and meta-analysis on pretreatment plasma D-dimer's prognostic role in solid tumor patients [25]. Their study provided strong evidence that increased pretreatment plasma D-dimer levels correlated with an unfavorable OS. They observed that the correlation between the elevated D-dimer level and the adverse prognosis remained considerable in various clinical cancer environments, ethnicities, with different detection methods and cut-off values. Thirty-seven studies, including a total of 10,176 patients, were used to provide multivariable analysis of hazard ratios (HRs) for OS. A high pretreatment D-dimer was significantly associated with worse OS in a random-effects model (pooled HR = 1.90, 95% CI = 1.63-2.20, P < 0.001) with substantial heterogeneity among studies (I2 = 75.1%, P < 0.001).

Some researchers claim that D-dimer influences cancer patients' prognosis through the development of venous thromboembolisms (VTEs). VTEs are a common cancer complication [26]. Overall, the risk of VTE in cancer patients is as high as 7%, possibly due to prothrombotic effects of malignant tumors and treatment-related risk factors such as immobilization, medications, and surgery [27]. Ay et al. stated that, according to their large-scale prospective results obtained, the correlation of D-dimer and VTE with an unfavorable cancer prognosis were independent of one another in solid tumors [28]. They clarified this result because abnormally elevated D-dimer could be the product of a downstream reaction from the extrinsic pathway of the coagulation cascade.

Blackwell et al. stated that elevated D-dimer levels in cancer patients might result from increased fibrinolytic activity levels [29]. Clinical studies in cancer patients' care with anticoagulation therapy have been performed. Findings have indicated that anti-thrombotic agents, such as warfarin and low molecular weight heparin, are successful in preventing and treating hematogenous metastases and survival prolongation [30, 31]. Dai et al. have shown that increased plasma levels of D-dimer have been linked with clinical cancer stages and metastases in patients with breast, gastric, colon, and rectal cancer [32]. They identified an association between plasma D-dimer levels and pancreatic cancer, and gender in colon cancer patients. These results indicate that the D-dimer level has potential use in determining the risk of metastases and development of multiple different cancers.

Diao et al. reported a marked increase in plasma D-dimer levels in gastric cancer patients with distant metastases, especially in patients with visceral metastases [33]. They also proposed the use of D-dimer in the clinical staging of patients with gastric cancer. Lee et al. stated that increased plasma D-dimer and fibrinogen degradation levels were substantially correlated with the TNM stage in colorectal cancer patients [34]. Preoperative plasma D-dimer levels have also been associated with elevated tumor size and lymph node metastases in colorectal cancer patients [10]. Additional studies have shown that the level of plasma D-dimer in pancreatic cancer patients may predict OS and progression-free survival [35]. Lin et al. confirmed that elevated D-dimer levels predicted a worse prognosis in patients with digestive cancers [36]. Their research offers compelling evidence that D-dimer is a significant and prominent tumor biomarker in digestive cancers and has a more robust potency in prognosis prediction.

Despite advancements in medical care, women with metastatic breast cancer have a poor prognosis, with a low OS rate of two to three years [37]. In patients with TNM stage III-IV disease, plasma D-dimer levels were substantially higher than those in patients with stage I-II disease. In patients with lymph node metastasis, plasma D-dimer levels were also higher than in patients without metastasis. A progression of the condition, a later clinical stage, and a greater risk of tumor metastasis are indicated by elevated D-dimer levels. Plasma D-dimer levels can be used for the diagnosis and staging of breast cancer as an auxiliary index. Increased D-dimer levels have been associated with tumor growth rate, tumor burden, progression rate, and survival [38]. D-dimer positively correlates with tumor size and lymph node metastasis. The correlation with tumor characteristics may indicate that the primary tumor is imprinted after resection on the coagulation system and may also represent residual occult circulating tumor cells' activity on blood coagulation. Breast cancer cells can cause blood coagulation by several mechanisms [39]. In vivo studies in patients with breast cancer have shown significant associations between increased D-dimer levels with circulating tumor cells, invasion of lymphatics or blood vessels, and clinical stage [22].

The mechanism underlying the link between elevated levels of plasma D-dimer and malignancy remains theoretical. One study reported that tumor cells directly stimulated the coagulation system, disrupted the endothelial vascular wall's intactness, and increased platelet and fibrinolytic protein activity [40]. Further research is required to clarify the biological mechanism of how high D-dimer impacts the development of malignancy in cancer patients.

Limitations

The current study was limited in that it only identified the association of D-dimer with cancer. The D-dimer level can be influenced by many factors such as age, sex, tumor stage, and coagulopathies. These confounders were not assessed in this study.

## Conclusions

Cancer has an enormous negative impact around the world. Early detection of cancer, cancer recurrence, and advanced cancer screening methods can help treat cancer, detect recurrence, and reduce national spending. D-dimer plasma levels were significantly higher in patients with cancer and associated with clinical stages and metastasis. There is evidence that elevated pretreatment plasma D-dimer is associated with adverse survival among patients with different solid tumors. Analysis of the pretreatment plasma D-dimer might provide useful information to predict prognosis in patients with solid tumors.

This study may have drawbacks, but D-dimer still seems to be promising in diagnosing and predicting prognosis in cancer patients. Further research is required to examine D-dimer's sensitivity and specificity in cancer screening and as a marker for recurrence of cancer. Further observational and intervention studies are also required to determine if plasma D-dimer levels can be integrated into the cancer staging system. Standardization and consistent communication of the performance characteristics of D-dimer would enable the test to be used successfully. D-dimer levels can be integrated into prognostic models. Additionally, more research should be performed to illustrate the association between high D-dimer levels and tumor progression.
